# Laparoscopic liver resection: indications, limitations, and economic aspects

**DOI:** 10.1007/s00423-020-01918-8

**Published:** 2020-07-01

**Authors:** Moritz Schmelzle, Felix Krenzien, Wenzel Schöning, Johann Pratschke

**Affiliations:** Department of Surgery, Campus Charité Mitte and Campus Virchow-Klinikum, Charité - Universitätsmedizin, Corporate Member of Freie Universität Berlin, Humboldt-Universität zu Berlin, Augustenburger Platz 1, 13353 Berlin, Germany

**Keywords:** Laparoscopic liver surgery, Minimally invasive liver surgery, Robotic liver surgery

## Abstract

**Background:**

Minimally invasive techniques have increasingly found their way into liver surgery in recent years. A multitude of mostly retrospective analyses suggests several advantages of laparoscopic over open liver surgery. Due to the speed and variety of simultaneous technical and strategic developments, it is difficult to maintain an overview of the current status and perspectives in laparoscopic liver surgery.

**Purpose:**

This review highlights up-to-date aspects in laparoscopic liver surgery. We discuss established indications with regard to their development over time as well as continuing limitations of applied techniques. We give an assessment based on the current literature and according to our own center experiences, not least with regard to a highly topical cost discussion.

**Conclusions:**

While in the beginning mainly benign tumors were laparoscopically operated on, liver metastasis and hepatocellular carcinoma are now among the most frequent indications. Technical limitations remain and should be evaluated with the overall aim not to endanger quality standards in open surgery. Financial aspects cannot be neglected with the necessity of cost-covering reimbursement.

## Introduction

Laparoscopic surgery of parenchymatous organs, e.g. liver and pancreas, has been developed relatively late, even though first reports of minimally invasive liver resections were back in the 1990s [1]. The development of laparoscopic liver surgery was accompanied by reservations and questions that have already been cleared or clarified years ago in other areas, such as colorectal surgery. However, as liver surgery differs significantly in many respects, it was not simply a matter of adopting answers from other fields, but of putting the feasibility and usefulness of the laparoscopic technique to the test again. The development has benefited from an early exchange between international pioneers in this field. The organization of consensus and guidelines meetings, e.g., 2008 in Louisville, USA, 2014 in Morioka, Japan, 2018 in Southampton, UK, and later the founding of the International Laparoscopic Liver Society (ILLS), which organized first meetings in Paris in 2017 and Tokyo in 2019, were instrumental in the successful development of safe laparoscopic liver surgery [2–4].

Besides the pioneering spirit of some, its implementation was only made possible by technical innovations and strategic modifications of open surgery. While minimally invasive surgery has long been used equivalently for laparoscopic surgery, different techniques and strategies have emerged from autodidactic learning. Simultaneously, experience was gained in multiport, single-port, hand-assisted, and hybrid techniques, whereby country-specific preferences developed [5, 6]. Intraoperative strategies, e.g., the caudal approach, and ideal techniques of parenchymal dissection were dominant topics in those days [7, 8]. Experiences with different entities, such as hepatocellular carcinoma (HCC) in cirrhosis, also varied greatly from country to country, depending on the local incidence and alternative treatment options, such as liver transplantation [9]. Since the first ILLS meeting in Paris in 2017, we have recently seen a homogenization of indications, applied techniques, and strategic approaches in laparoscopic liver surgery.

## Indications

### Benign liver tumors

Symptomatic benign liver tumors (BLT), e.g. focal nodular hyperplasia, hemangioma, or hepatocellular adenoma, were the first to be resected laparoscopically back in the 1990s and early 2000s [10]. It became clear early on that the technique inherited certain advantages compared with the conventional open procedure. Overall morbidity, time to oral intake, and hospital stay were repeatedly shown to be reduced after laparoscopic liver surgery compared with the conventional open technique [11]. The relatively high rate of conversions to open surgery was not surprising as the management of intraoperative bleeding in particular was still a major challenge in those days [11]. All the more important for the successful implementation of this technique was the fact that there was no mortality in first multicentric reports [11]. It further simplified the implementation that no oncological concerns had to be eliminated with these resections in benign lesions. Meanwhile, retrospective studies with propensity score matching (PSM) and systematic reviews have proven the feasibility and safety of the minimally invasive technique for BLT, independent of the location, complexity, and resection extend [10–12]. Since prospective studies are probably not to be expected due to the rare indication alone, the laparoscopic technique can be defined as standard of care for BLT on the basis of the available data. Symptomatic growth is one of the indications for the resection of BLT and the question of whether the quality of life (QoL) can further be improved by the laparoscopic approach is another interesting aspect. In a recent review of 42 studies, eight studies used validated QoL tools. Of these eight studies, only two could show an advantage for laparoscopic over open surgery. Consequently, the superiority of the laparoscopic technique in terms of increasing QoL must be considered controversial, at least for BLT, and one must be careful in drawing any obvious conclusions [13].

### Colorectal liver metastasis

After liver metastases could already be diagnosed laparoscopically in the 1960s, first reports of laparoscopic partial resections of superficially located and easily accessible colorectal liver metastases (CRLM) date back to the 1990s [14, 15]. However, the time for a broad application of laparoscopic liver resections in CRLM has not yet come in the late 1990s since other questions remained unanswered, such as the reliable detection of occult liver metastases [16]. Consequently, the potential added value of minimally invasive techniques in CRLM was evaluated primarily with a view to improving staging and allowing appropriate selection of patients for resection, e.g. by diagnostic laparoscopy and laparoscopic ultrasonography [17]. Brice Gayet from Paris was one of the first to publish larger case series including laparoscopic major anatomical resections in CRLM in 2004 [18]. Even though conversion and complication rates were still comparatively high, acceptable oncological results were achieved in CRLM [18]. More than 10 years later, systematic reviews and meta-analysis including 4591 and 2259 patients, respectively, confirmed favorable short-term outcomes after laparoscopic liver surgery for CRLM with significantly reduced blood loss, lower operative transfusion requirement, shorter hospital stay, reduced overall morbidity, and reduced severe morbidity compared with conventional open surgery [19, 20]. Regarding oncologic outcomes, there were no significant differences found between laparoscopic and open surgery, except for a higher R0 resection rate in the laparoscopic group. The latter rather confirmed the critics who attributed the convincing results to a selection in favor of laparoscopically operated cases. Thus, a randomized controlled trial (RCT) was urgently demanded. The first RCT (OSLO-COMET) comparing laparoscopic and open resections in CRLM was published 1 year later—in 2018—by Fretland et al. [21]. Two hundred eighty patients were randomized, of whom 144 open and 129 laparoscopically operated patients were analyzed in the final evaluation. The results confirmed clear advantages for the laparoscopic procedure. Significant differences were found with regard to lower postoperative pain medication requirement, lower complication rate (Dindo-Clavien > II), lower comprehensive complication index, and shorter intensive care unit and hospital stay. In addition to the advantages already mentioned above, a subgroup analysis of the OSLO-COMET group using a standardized questionnaire revealed a higher QoL for patients after the resection of posterosuperior segments [22]. Long-term oncological results of this first RCT in laparoscopic liver surgery are still pending, but the results of previous meta-analyses and retrospective PSM analyses let anticipate favorable outcomes, at least a non-inferiority for the laparoscopic approach.

### Strategies in colorectal liver metastasis

CRLM are often arranged multilocular and bilobar. Since they usually arise in normal liver parenchyma, except for chemotherapy-associated alterations, all strategic options must be considered when surgical treatment is planned [23]. Two-stage hepatectomy is a concept that has been established for more than 20 years with a large number of technical variants, which can help to address metastases initially classified as unresectable [24]. According to a French retrospective multicenter analysis of about 2500 patients with CRLM from 2015, almost 20% of laparoscopically resected patients had bilobar lesions [25]. Portal vein embolization (PVE) as part of the concept was necessary in 13 of the 176 patients (7.4%). The proportion of patients with extensive findings was lower than in the group of patients undergoing open surgery, clearly indicating a selection bias. Nevertheless, the authors concluded that patients who were considered suitable for laparoscopic liver resection appeared to benefit from the minimally invasive approach without impairing long-term survival. These results were confirmed in a recent study. Okumura et al. analyzed retrospective data from two experienced centers in Paris and confirmed laparoscopic two-stage hepatectomy in bilobar CRLM to be safe and associated with an improved outcome compared with open surgery [26]. In addition to established concepts such as PVE, hypertrophy of the left hepatic lobe can also be safely achieved by minimally invasive ALPPS (Associating Liver Partition with Portal vein ligation for Staged hepatectomy) with supposed advantages over the open technique [27]. Speaking from personal experience, ALPPS can be performed safely laparoscopically as well as robotic-assisted, although equivalence must be confirmed by future comparative studies.

### Hepatocellular carcinoma and liver cirrhosis

HCC is considered the most common indication for laparoscopic liver resection besides CRLM and results in HCC seem to confirm those in CRLM. First experiences in laparoscopic liver resections for HCC go back over 20 years as reported by Daniel Cherqui in a feasibility study in 30 patients [28]. Meanwhile there is one controlled randomized study (*n* = 25 vs. *n* = 25) comparing laparoscopic and open liver resection in early HCC (< 5 cm) in compensated cirrhosis (CHILD A) from Egypt [29]. Operative time and hospital stay were decreased in the laparoscopic group with otherwise comparable perioperative results. The further course of the patients suggested that the oncological result was not affected by the laparoscopic procedure. Due to several limitations, this study must be evaluated with caution. Nevertheless, it should be mentioned that these results were supported by PSM analyses from Asia (Korea [30] (*n* = 33 vs. 33), China [31] (*n* = 32 vs. 32), Hong Kong [32] (*n* = 110 vs. 110)) and our group in Berlin [33] (*n* = 56 vs. 56). Repetitively, an advantage of the laparoscopic approach could be shown, especially with regard to a decrease of intra- and postoperative complications. Besides perioperative advantages, these analyses also confirmed a comparable oncological outcome with at least equivalent overall and recurrence-free survival. Although it has not yet been shown to be pathophysiologically conclusive, reduced trauma of the abdominal wall with less resulting pain and the preservation of collaterals seem to have a more significant beneficial influence than in liver healthy patients. As advantages can be witnessed especially in those with advanced cirrhosis (CHILD B), surgical indications may be extended in cirrhotic patients by advocating the minimally invasive approach even in unfavorable segments [34, 35]. It seems as if the underlying cirrhosis of the liver speaks clearly for and not against the minimally invasive technique, as initially postulated by some. This is supported by the 2018 EASL Clinical Practice Guidelines on the Management of Hepatocellular Carcinoma, according to which optimal surgical candidacy is based “on a multiparametric evaluation including compensated Child-Pugh class A liver function with MELD score <10, to be matched with grade of portal hypertension, acceptable amount of remaining parenchyma and possibility to adopt a laparoscopic/minimally invasive approach” [36].

### Intrahepatic cholangiocarcinoma

First case reports in laparoscopic liver resections for intrahepatic cholangiocarcinoma (iCC) also go back ~ 20 years [28, 37]. Harimoto et al. reported in 2002 on a left lateral sectionectomy with hilar lymphadenectomy (LAD) and recognized possible advantages of the minimally invasive technique in reduced blood loss and shortened hospital stay [37]. The low number of publications on minimally invasive liver resections in iCC in the following years was not only due to rarity of those tumors. In fact, the necessity of extended liver resections with/without biliary reconstruction and the complexity of hilar LAD have so far prevented a broad acceptance of laparoscopic techniques in iCC. Nevertheless, a recent systematic review of four retrospective observational studies including 204 patients with iCC basically confirmed results in CRLM and HCC [38]. Laparoscopic liver resection for iCC appears to provide short-term benefits without negatively affecting oncologic adequacy with regard to microscopically tumor-free margins and disease recurrence [38]. These results can be traced by means of a recent analysis from Berlin [39]. Clinical courses of all consecutive patients (*n* = 159) undergoing liver resection for iCC between January 2015 and October 2019 were reviewed, of which 23% of the interventions were performed minimally invasive [39]. After applying selection criteria, oncological short-term results after laparoscopic liver resection met oncological demands of open surgery with a R0 resection rate of 89% (74% in open surgery) and a median number of eight retrieved lymph nodes (*n* = 8 in open surgery), latter complying with the AJCC staging manual (8th edition of American Joint Committee on Cancer) of 2017. A decreased overall morbidity (Dindo-Clavien 1–5) of 30% in laparoscopic compared with 58% in open surgery translated in a shortened hospital stay of 8 days vs. 10 days, respectively. Obviously, our study should to be interpreted with caution due to the retrospective study design and high rate of excluded patients alone [39]. The majority of patients were apparently identified as unsuitable for the laparoscopic approach. Nevertheless, the study suggests that a selection of patients with iCC is reasonably possible and laparoscopic liver surgery in iCC should no longer represent a general contraindication.

## Limitations

### Difficulty scores indicate possible limitations

There are multiple definitions and risk scores available to assess the difficulty of laparoscopic liver resections, of which the Iwate criteria are most commonly used [40–43]. Several parameters are included in the Iwate criteria, such as tumor location, tumor size, proximity of the tumor to large vessels, liver function, and technical considerations. All these aspects, alone or in combination, are usually not a contraindication per se, but can well be limiting with regard to the laparoscopic approach. Those criteria may help to define limitations individually depending on the findings and the personal as well as center-specific learning curves. Interestingly, not only the difficulty of a procedure can be assessed by the Iwate criteria from those preoperative variables but also postoperative complications [41]. This is of importance as possible limitations should be defined not only by technical restrictions but also by the rate of postoperative complications and the adherence on high (oncological) quality standards in open surgery.

### Abdominal adhesions and repeat liver resections

Abdominal adhesions develop regularly after surgery and in particular after conventional open interventions. The extent of adhesions depends on the procedure and the postoperative course with significant interindividual differences in patients. Abdominal adhesiolysis can sometimes be technically complex and extensive adhesions often require adhesiolysis for several hours, even in the conventional open technique. According to a recent multicentric analysis of 2856 patients being scheduled for laparoscopic liver resection, previous open liver resection was found to be an independent predictor of intraoperative complications [44]. Indeed, extensive adhesions can be limiting for the laparoscopic approach, especially when evident in the upper abdomen, and the risk should be carefully weighed when choosing the technique. However, scars do not automatically indicate extensive adhesions. Even though assumptions are certainly slightly distorted by a preoperative patient selection, the rate of patients who cannot be operated laparoscopically due to prior open surgery is expected to be in the lower percentage range. As recently shown, almost half of our patients being scheduled for laparoscopic liver resection had a history of previous abdominal interventions [45]. Neither the postoperative complication rate nor the hospital stay was affected by abdominal adhesions in those patients. The above also applies to repeated liver resections, as recently shown for both CRLM and HCC [46, 47]. If repeated liver resection is considered possible laparoscopically, patients have common advantages over the open technique, e.g., less blood loss, a lower complication rate, and reduced hospital stay.

### Giant tumors

As Shelat et al. could show, laparoscopic liver resection is feasible and safe for large malignant tumors and can be performed with an acceptable morbidity and oncologic efficiency [48]. However, the subgroup analysis of “giant” malignant tumors indicated that LLR is associated with greater blood loss and a longer operative time. Accordingly, the size of the tumor is also taken into account in the Iwate criteria, so that tumors larger than 3 cm are classified with an increase in difficulty [40]. Giant tumors in particular are extremely demanding to operate on, where limits of what is considered technically feasible can easily be reached. The placement of trocars, the mobilization of a liver lobe, and the accidental tumor perforation by shear forces are examples of possible intraoperative difficulties. Further arguments against the laparoscopic procedure for giant tumors include that conventional recovery bags are too small and a comparatively long incision is required to retrieve the specimen. In this respect, the question of feasibility depends primarily on whether the resection can be performed safely and within a reasonable time. Of note, if technically feasible, well-known advantages of minimally invasive surgery have also been confirmed in the literature for giant tumors [49]. Therefore, the decision whether to operate laparoscopically or rather conventionally open should be based on the findings and, again, on the own learning curve and personal experiences made.

### Multiple bilobar liver metastases

Besides giant tumors, the role of laparoscopic liver surgery is also controversial in case of multiple bilobar metastasis. As mentioned above, according to a recent French study, two-stage hepatectomy in bilobar CRLM is obviously safe and associated with an improved outcome compared with open surgery [26]. As Okumura et al. reported, number and maximum diameter of lesions at first stage ranged from 1 to 5 and 3 to 40 mm after propensity score matching, respectively [26]. Of note, microscopically tumor-free margins could be achieved in 96% [26]. These data are indeed convincing and speak for the long experience of these centers in complex laparoscopic liver surgery. Without any question, a broad experience in sonography-guided parenchymal dissection is indispensable [50]. 3D laparoscopy, as is standard practice at the Hôpital Pitié-Salpêtrière in Paris, should be mentioned as an another example for important supportive visual techniques. Use of indocyanine green (ICG), being widely used in Japan and experienced centers elsewhere, can further help to both detect superficial lesions and guide difficult parenchymal dissection [51]. Research is being conducted on the application of virtual realities, what could also be beneficial in this context [52, 53]. Nevertheless, multiple partial resections of (superficial) liver metastases remain technically demanding without all these aids and extremely time-consuming, e.g., in case of multiple vanishing lesions after chemotherapy. In this respect, one must also make a good estimate of which efforts are really useful in everyday clinical practice.

### Resection of posterosuperior segments

The standard positioning of the optic trocar provides a view from caudal to cranial. In this regard, the so-called caudal approach has proven itself in several laparoscopic resections, e.g., right or left hepatectomy [54]. As much as this strategy may be useful for caudocranial parenchymal dissection, it is of little use for the resection of posterosuperior segments. Especially the anatomical resection of segments 7 and 8 (dorsal) can well be borderline feasible due to a poor overview and unfavorable working angles. In recent years, approaches have been adapted exemplified by the appropriate positioning of the patient and the intercostal placement of trocars [55]. Further, dissection strategies have been refined, e.g., Diamond technique, allowing safe laparoscopic parenchymal-sparing liver resections of lesions in posterosuperior segments, even in cirrhosis [56, 57]. Nevertheless, these interventions often represent limitations and should be reserved for most experienced laparoscopic liver surgeons, who can safely master possible complications such as (venous) bleeding, e.g., from the right hepatic vein [58]. It is not for nothing that resection of segments 7 and 8 is rated with a maximum score of 5 in the Iwate criteria [40]. The overall goal must always be a safe resection that fulfills high oncological quality criteria of open surgery. Compromises in favor of the laparoscopic technique should be viewed critically and early conversion in case of suboptimal settings, e.g., to hand-assisted or hybrid liver resection, should be preferred.

### Hilar lymphadenectomy

As recently reported, fully laparoscopic dissection of hilar lymph nodes is considered technically possible and seems safe in experienced hands [39, 59]. This is noteworthy as technical limitations in hilar LAD were thought to be a contraindication to laparoscopic surgery for iCC in the past. General expectations were dampened by first reports and doubts regarding the oncological equivalence of the minimally invasive procedure arose. The median number of retrieved lymph nodes often did not correspond to current recommendations for iCC [38, 60]. Moreover, cases of laparoscopically operated iCC in which no LAD was performed at all were disturbing [38, 60]. In a US national cancer database analysis of 2309 patients with iCC, the percentage of nodal evaluation was significantly lower in patients undergoing laparoscopic liver resection (39%) compared with patients being scheduled for open surgery (61%) [61]. Given an overall nodal evaluation of 58%, the experience of reporting centers seems particularly important, not only in laparoscopic surgery [61]. Therefore, the discussion about possible limits of LAD is very well suited to describe the further development of laparoscopic (and open) liver surgery in the coming years in general. We will likely see a development towards a broad application of laparoscopic surgery for technically less demanding liver lesions with highly specialized applications, such as extended hepatectomy with LAD, being reserved for a few centers with the appropriate experience. To accompany this twin-track development, strict quality control is required to ensure equivalence to the conventional open technique. A first step in this direction would be to include hilar LAD in revised risk scores in the future.

### Biliary reconstruction

According to a current publication from Germany, mortality after liver resection increases depending on the extent and need for biliary reconstruction up to 25% [62]. Trisectionectomy with hepaticojejunostomy is a time-consuming and technically most demanding intervention even in the conventional open technique. Possibilities of reconstructing small bile ducts to jejunum are clearly limited in the laparoscopic technique that, apart from the quality achieved, would likely further extend the operating time. The question arises whether open surgery should be reserved for these particularly complex interventions. In retrospect, there were many technical boundaries that were torn down one after the other. Therefore, it is not surprising that in the meantime, there are first case series of minimally invasive surgery for perihilar cholangiocarcinoma (PHC) [63–65]. Evidence has been provided that both extended hepatectomy as well as biliary reconstruction should be considered technically possible. However, these reports are not sufficient for a robust assessment. Experience with PHC is up to now limited to individual cases and small case series from highly experienced centers [63, 65, 66]. Larger series or ideally comparative studies are not to be expected in the near future due to the rarity of these interventions alone. Improvements to technical possibilities, e.g., a simplification of sewing, are needed to sufficiently overcome this last hurdle. Robotic-assisted liver surgery is still in its infancy and first experience does not yet allow a final evaluation. From our personal experience, however, first reports can be traced after the robotic technique could possibly have advantages in sewing and thus biliary reconstruction [67, 68].

### Living donor liver transplantation

Repeatedly shown perioperative advantages of the laparoscopic technique, e.g., reduced blood loss and lower complication rates, seem particularly attractive in healthy individuals as for both pediatric and adult living donor liver transplantation (LDLT). Indeed, there are an increasing number of studies suggesting favorable postoperative outcomes after laparoscopic donor hepatectomy for LDLT. Especially laparoscopic left lateral sectionectomy is meanwhile a standard procedure in specialized units yielding expected advantages in the donor with comparable short and long-term outcome to open surgery in the recipient [69–71]. However, a learning curve of 25 fully laparoscopic living donor left lateral sectionectomies under proctorship for an expert liver transplantation surgeon was recently suggested, which indicates that safe introduction of laparoscopic minor liver resections is restricted to high volume LDLT centers [72]. The significance of major liver resection in LDLT has not yet been conclusively clarified [73]. Results from a recent series of 412 minimally invasive donor hepatectomies being performed at 10 leading centers were comparable with those of benchmarking series of open standard donor hepatectomy with a morbidity below 10% (Dindo-Clavien 3–4) and no mortality [74]. A retrospective analysis of 123 pure laparoscopic donor right hepatectomies (PLDRH) confirmed similar complication rates to the open technique [75]. However, there was a trend towards more biliary complications (leak and stricture) compared with the open technique (*n* = 123) after propensity score matching. This underlines the importance of a previous study reporting on a higher percentage of multiple bile duct openings in PLDRH [76]. With regard to the course of the recipient, it seems noteworthy that time to remove the liver and warm ischemic time are usually longer after PLDRH compared with open right hepatectomy [77]. This is clinically significant because the longer ischemic time must be blamed, at least in parts, for the higher rate of biliary complications after PLDRH [77]. A final evaluation of this technique is not yet possible based on the available data and PLDRH remains controversial due to safety concerns both in the donor and in the recipient. More experience needs to be gathered especially on long-term outcomes and with regard to biliary complications.

### Hybrid and hand-assisted techniques

The use of hybrid and hand-assisted techniques (HALS) leads to a decrease in complexity in the Iwate criteria [40]. This is of importance as individual technical limits can easily be shifted by using these techniques. Especially in the learning curve hybrid and HALS can be useful and allow complex resections that would otherwise be hardly possible at this stage [78]. In a recent Italian series of PHC, biliary reconstruction after extended hepatectomy using hybrid techniques completed a previously fully laparoscopic resection [64]. Even though the insertion of a handport may be associated with an increased incidence of hernias in the long term, the postoperative outcome does not seem to be negatively affected, when compared with fully laparoscopic surgery [78, 79]. From a personal perspective, the liberal combination of different techniques will be trend-setting rather than the compulsive desire to adhere to fully laparoscopic surgery. This will also apply to robotic-assisted surgery, which will complement rather than replace established minimally invasive techniques, striving to expand indications while maintaining oncological principles and to reduce complications [80].

## Economic aspects

With regard to economic aspects, the comparison of laparoscopic and open liver surgery is particularly difficult. One must distinguish precisely between minor and major liver resections, intraoperative and postoperative costs, and country-specific reimbursement systems. One has to weigh up classical cost drivers, such as postoperative complications, against new ones in laparoscopic surgery, such as expensive stapler cartridges. A distinction must also be made between real and virtual costs, e.g., for human resources, and these must be constantly evaluated in the light of modified reimbursement systems.

### Intraoperative costs

In a retrospective analysis, our group compared costs in laparoscopic and open hemihepatectomy being allocated to the DRG (diagnosis-related group) H01A (complex operations of the liver and pancreas with complex intensive care treatment) or H01B (operations of the liver and pancreas without complex intensive care treatment) [81]. After propensity score matching, intraoperative costs were significantly higher in laparoscopic compared with open hemihepatectomy with ~ 8000€ and ~ 4000€, respectively. In fact, the difference in intraoperative costs between both groups was mainly due to the laparoscopic instruments with 73% and a longer operation time with 27%. Higher total intraoperative costs were confirmed by a retrospective analysis from UK [82] and USA [83] and the prospective OSLO-COMET trial from Norway [21]. Whereas consumables/disposable equipment were considered the main cost driver in minor resections, costs for staff and theater time are obviously gaining in importance in laparoscopic major hepatectomy [21, 82]. Importantly, intraoperative costs can possibly even be reduced in minor resections by a shorter operation time when compared with open surgery [82].

### Important intraoperative cost drivers

The extended operating time is a decisive disadvantage of the laparoscopic technique in terms of cost-effectiveness. However, it should not be forgotten that the data generated so far have been collected within the framework of learning curves and an improvement in this respect is expected in the coming years at many centers [84, 85]. Staplers are commonly used in laparoscopic liver surgery for transection of parenchyma and vessels; however, they are among the major cost drivers in laparoscopic surgery [81, 86, 87]. Therefore, econometric aspects should not be disregarded when selecting intraoperative strategies and favored techniques. Anyways, prices of laparoscopic devices will likely decrease in the future due to the increasing demand. Increasing competition from manufacturers and expiring patent rights will create further cost pressure, so that these aspects will fade into the background.

### Postoperative and total costs

Liver resection in general is still associated with a high incidence of complications that lead to a considerable financial burden. Therefore, low complication rates of laparoscopic liver surgery may be favorable for billing [88]. In our analysis, patients after laparoscopic hemihepatectomy were indeed characterized by a significantly lower rate of postoperative complications and a shortened median hospital stay of 5 days [81], resulting in comparable total costs after laparoscopic and open hemihepatectomy with 17,369.85€ and 16,103.64€, respectively. Cost-effectiveness was confirmed by the OSLO-COMET trial due to shorter stays on ICU and hospital stay and a lower readmission rate [21]. Abu Hilal et al. from UK showed that the lower postoperative costs for both major and minor procedures are mainly due to cost reduction on ICU, wards, bloods and X-ray [82]. This resulted in same total costs in major hepatectomy and even lower total costs after left lateral sectionectomy. Cost-effectiveness of laparoscopic resections obviously varies with the complexity of the procedure. Higher intraoperative costs, e.g., for surgical equipment or due to longer operating times, can obviously be recouped by a smaller deviation from the normal postoperative course, with advantages noted especially for laparoscopic minor resections.

### Comparability of different centers

Costs can be easily calculated as shown above; however, different currencies (e.g., USD and Euro) and reimbursement systems make an international comparison hardly possible. Not even the one-off cost analysis of single centers may provide a profound basis for the upcoming years. Due to constantly changing costs and modifications in reimbursement systems, a constant update of the calculation is necessary. The surgeon is thus jointly responsible for ensuring that this new technique is not withheld from patients due to financial considerations. In addition, more work will be needed in the future to adapt the reimbursement for the laparoscopic technique accordingly. The country of Japan should be noted in this regard as insurance reimbursements are guaranteed for all types of laparoscopic liver surgery (without vascular and biliary reconstruction); likewise, the insurance companies demanded a mandatory registry database. The number of LLRs has increased remarkably in Japan since the introduction of this special refund [89].

### Costs in robotic-assisted liver surgery

In a recent study from the USA, overall benefits of minimally invasive liver surgery could be confirmed for robotic-assisted liver resection (*n* = 68) with fewer complications, shorter ICU, and hospital stay compared with open liver resection (*n* = 55) [90]. This resulted in comparable average total costs with $37,518 for robotic surgery and $41,948 for open technique. The cost-effectiveness of robot-assisted liver resection compared with conventional laparoscopic liver resection, however, has hardly been investigated so far. Should robotic-assisted surgery find its way into liver surgery in the long term, it will be necessary to discuss costs and reimbursement at an early stage. In particular, it must be clarified in this context which interventions should be performed laparoscopically and which robotic-assisted. It also remains interesting to see how the costs develop after new providers have entered the market.

## Summary

In recent years, liver surgery has been increasingly integrated into multimodal therapeutic strategies. In this respect, the focus has been on the safety of surgical interventions and low complication rates. These growing quality demands have paved the way for gentle, minimally invasive techniques into complex liver surgery. There are now a large number of mostly retrospective studies that have repeatedly confirmed intra- and postoperative advantages over conventional open liver surgery. Initial fears that this would have to be weighed against the oncological disadvantages of minimally invasive techniques were not confirmed based on existing studies. In this respect, it is currently necessary to evaluate different minimally invasive techniques with regard to their advantages and possible weaknesses.

CRLM and HCC in cirrhosis have proven to be particularly suitable for laparoscopic approaches, and initial prospective studies have now confirmed this for these entities. While CRLM and HCC together account for more than half of the indications at most centers, minimally invasive surgery of CCA is still limited to a small number of centers with very high expertise. Limitations in general and in CCA in particular often result from the complexity of interventions, some of which can be estimated by means of established complexity scores. However, compromises with regard to intraoperative safety and the adherence to oncological principles need to be considered inacceptable. In this respect, the risk of a laparoscopic resection must always be assessed on the basis of the surgeon’s own learning curve and individually weighed against the potential benefit for the patient. As some limitations of minimally invasive techniques will likely remain in the foreseeable future, open surgery can only be supplemented but not replaced.

Despite all optimism in this field, economic considerations in laparoscopic and robotic-assisted liver surgery cannot be ignored. Laparoscopic liver surgery is generally associated with an increased cost for intraoperative consumables and, at least in the learning curve, longer operating times. As higher intraoperative costs are offset by lower postoperative costs, comparable total costs often result. A holistic understanding of costs and charges in laparoscopic and open liver surgery seems particularly important in discussions with hospital administration. With regard to minimally invasive and especially robotic-assisted liver surgery, it therefore seems essential to describe the advantages and substantiate them as thoroughly as possible. The necessity of cost-covering accounting must also be made clear, analogous to lived realities in Japan. Since there is no culture of negative reporting in the field of surgery, it seems reasonable to adopt efforts for quality control from countries like Japan with the introduction of mandatory nationwide registries.
